# Dr. Gregory's Reply to the Animadversions of Medicus on His Paper on Constitutional Inaptitude to Cow-Pox

**Published:** 1828-01

**Authors:** 


### Dr. Gregory's *Reply to the Animadversions of* Medicus *on his Paper on Constitutional Inaptitude to Cow-Pox.*

Your correspondent at Cheltenham is pleased to express his "surprise" at the request I made in a former Number of the Journal to be put in possession of the peculiar opinions entertained by Dr. Jenner on the subject of a constitutional inaptitude to cow-pox; and he cannot conceal his "astonishment" that I should not have known that they had been for several months before the world in an authentic form; that is to say, in the seventh chapter of Dr. Baron's Life. If such were his feelings then, I dread to think what they will be now, when I confess that I am still as ill informed as before, and that all I have gained from his remarks is the shrewd suspicion that he is in the same condition of ignorance with myself. If the subject had indeed been discussed in Dr. Baron's work, it would have been an easy task to have pointed at once to the exact page where it may be found. But no, sir, your correspondent favors us instead with some remarks on a speculative question totally different from the practical one at issue, and follows this up by a process of reasoning which, as he states, will furnish us with the required truth. " Dr. Jenner," he says, " teaches the actual identity of (or at least the close affinity between) small-pox and cow-pox. Now, as there exists in some persons a constitutional unsusceptibility of small-pox, a like inaptitude might fairly, *a fortiori*, be predicated of cow-pox."

The force of this specimen of medical logic, which is, I dare say, quite conclusive in the eyes of your correspondent, is however entirely lost upon me. If, indeed, the case were reversed, and we were deducing the laws of small-pox from those of cow-pox, I might, perhaps, acknowledge it; just as I admit that a man who is incapable of walking ten miles cannot, *a fortiori*, walk twenty: but the merit of the argument that, because a man cannot walk ten miles, therefore, *a fortiori*, we cannot walk one, belongs exclusively to your ingenious correspondent. He probably meant to say, *pari ratione*, (which, though a loose mode of reasoning, is still intelligible;) but he who stands forward to correct the trippings of others should be careful not to stumble himself.

But, sir, there is another peculiarity in this train of reasoning, which struck me even yet more forcibly. Your correspondent, having, as we have seen, argued himself into a conviction of the strict analogy subsisting between the susceptibility of small-pox and of cow-pox, deduces thence the further conclusion that the laws of constitutional inaptitude to cow-pox are therefore known to us. This, sir, is, in my humble opinion, neither more nor less than what logicians call an argumentum *ab ignoto*, or *ad obscurius*. It attempts to explain one thing by reference to another, even less intelligible.

What are the laws of constitutional inaptitude to small-pox? At the risk of exciting the highest conceivable degree of astonishment in your correspondent's mind, I am free to confess that they are, in a great measure, unknown to me. Nay more, sir, I trust the time may never arrive when experience shall teach them to me; for I am as great a foe to inoculation as your correspondent at Cheltenham. That they were known to Baron Dimsdale and the professed inoculators of the old school, I can readily believe; and perhaps there may be some persons still engaged in practice to whom memory may make them familiar; but I think I can see, in the general tone of your correspondent's remarks, sufficient proof that he does not belong to that class.

The youngest of your readers can hardly have misunderstood me so far as to suppose that, when asking for Dr. Jenner's opinions regarding the unsusceptibility of cow-pox, I imagined him ignorant of the main fact that some constitutions were indisposed to it; but I wished to ascertain *how far* he had prosecuted this branch of the subject. To prevent all possible misunderstanding, however, in future, permit me to request specific answers from your correspondent Medicus to the following queries, with a hope that, instead of an argument *a fortiori,* he will favor me with the exact authorities (chapter and verse) on which his replies are founded.

1. Had Dr. Jenner any, and what, opportunities of witnessing a constitutional inaptitude to cow-pox existing *through life?*

2. Did we find the inaptitude to cow pox more or less prevalent than that to small-pox?

3. Did he observe that, for the most part, these conditions coexisted in the same individual?

4. Did he imagine that the inaptitude to cow-pox increased or diminished with the progress of life?

5. Could it, in his opinion, be obviated by any medical treatment?—and if so, by what means—whether purgatives or antimonials?

6. Did he consider that a constitutional inaptitude to cow-pox, clearly proved to exist for one or two years, authorised the adoption of variolous inoculation?

This, sir, is a mere sample of the questions now frequently put to me by persons both in and out of the profession, touching the constitutional inaptitude to cow-pox. Although holding what your correspondent is pleased to call the "highly responsible" office of physician to the Small-Pox and Vaccination Hospital, I candidly confess my inability to answer them satisfactorily. If your correspondent will supply the deficiency, with reference to authorities of easy access, I will then submit, and willingly acknowledge the justness of his animadversions, however harshly they may at first have sounded. In such a case, too, sir, I will take upon myself the blame of having occupied needlessly two pages of your valuable Journal.*

*8, Upper John-street, Golden-square;*
*December* 5, 1827.

* Should Medicus answer this "Reply," we hope he will have the goodness to do it in his own name.—Editor.

